# Copy number variation meta-analysis reveals a novel duplication at 9p24 associated with multiple neurodevelopmental disorders

**DOI:** 10.1186/s13073-017-0494-1

**Published:** 2017-11-30

**Authors:** Joseph T. Glessner, Jin Li, Dai Wang, Michael March, Leandro Lima, Akshatha Desai, Dexter Hadley, Charlly Kao, Raquel E. Gur, Nadine Cohen, Patrick M. A. Sleiman, Qingqin Li, Hakon Hakonarson, the Janssen-CHOP Neuropsychiatric Genomics Working Group, Patrick Sleiman, Joseph Glessner, Dexter Hadley, Charlly Kao, Zhi-liang Wu, Cecilia Kim, Kelly Thomas, Hakon Hakonarson, Dai Wang, Reyna Favis, Dong-Jing Fu, Hedy Chung, Adam Savitz, Srihari Gopal, Nadine Cohen, Qingqin Li

**Affiliations:** 1 The Center for Applied Genomics, Abramson Research Center, Children’s Hospital of Philadelphia, Philadelphia, PA 19104 USA; 2 Department of Cell Biology, Tianjin Medical University, Tianjin, China; 3 Janssen Research & Development, LLC, Raritan, NJ 08869 USA; 4 Department of Psychiatry, Perelman School of Medicine, University of Pennsylvania, Philadelphia, PA 19104 USA; 5 Department of Pediatrics, Perelman School of Medicine, University of Pennsylvania, Philadelphia, PA 19104 USA; 6 Division of Human Genetics, Children’s Hospital of Philadelphia, Philadelphia, PA 19104 USA; 7 Janssen Research & Development, LLC, Titusville, NJ 08560 USA

**Keywords:** Copy number variation, *DOCK8*, Gene-based analysis, Meta-analysis, Neuropsychiatric disorders, Quantitative PCR

## Abstract

**Background:**

Neurodevelopmental and neuropsychiatric disorders represent a wide spectrum of heterogeneous yet inter-related disease conditions. The overlapping clinical presentations of these diseases suggest a shared genetic etiology. We aim to identify shared structural variants spanning the spectrum of five neuropsychiatric disorders.

**Methods:**

We investigated copy number variations (CNVs) in five cohorts, including schizophrenia (SCZ), bipolar disease (BD), autism spectrum disorders (ASD), attention deficit hyperactivity disorder (ADHD), and depression, from 7849 cases and 10,799 controls. CNVs were called based on intensity data from genome-wide SNP arrays and CNV frequency was compared between cases and controls in each disease cohort separately. Meta-analysis was performed via a gene-based approach. Quantitative PCR (qPCR) was employed to validate novel significant loci.

**Results:**

In our meta-analysis, two genes containing CNVs with exonic overlap reached genome-wide significance threshold of meta *P* value < 9.4 × 10^−6^ for deletions and 7.5 × 10^−6^ for duplications. We observed significant overlap between risk CNV loci across cohorts. In addition, we identified novel significant associations of *DOCK8*/*KANK1* duplications (meta *P* value = 7.5 × 10^−7^) across all cohorts, and further validated the CNV region with qPCR.

**Conclusions:**

In the first large scale meta-analysis of CNVs across multiple neurodevelopmental/psychiatric diseases, we uncovered novel significant associations of structural variants in the locus of *DOCK8*/*KANK1* shared by five diseases, suggesting common etiology of these clinically distinct neurodevelopmental conditions.

**Electronic supplementary material:**

The online version of this article (doi:10.1186/s13073-017-0494-1) contains supplementary material, which is available to authorized users.

## Background

Neurodevelopmental and neuropsychiatric disorders represent a wide spectrum of heterogeneous yet inter-related disease conditions with significant overlap in phenotype expression. These diseases in children and young adults pose a major health burden and are growing in prevalence. As the clinical presentations of these diseases are not entirely distinct from each other, the clinical diagnostic boundaries are often hard to define [1].

Genome-wide association studies (GWAS) of single nucleotide polymorphisms (SNPs) in psychiatric diseases have begun to bear intriguing results [1–3]. Copy number variations (CNVs) have more direct gene dosage impacts and have been implicated in psychiatric diseases with larger effect size compared to SNPs [4–7].

Both GWAS and CNV studies have suggested different neuropsychiatric disorders share genetic determinants underlying disease development. It has been estimated that five major psychiatric disorders (schizophrenia (SCZ), bipolar disease (BD), autism spectrum disorders (ASD), attention deficit hyperactivity disorder (ADHD), and depression) share a degree of common genetic etiology. The Psychiatric Genomics Consortium (PGC) study exhibited a certain degree of genetic correlation which is relatively high between SCZ and BD, moderate between SCZ and depression, moderate between BD and depression, moderate between ADHD and depression, and non-zero between the other pairs of diseases, based on genome-wide SNP genotype data [3]. Common genetic loci have emerged from GWAS focusing on individual neuropsychiatric diseases [2, 8, 9]. Meta-analysis further suggested a shared genetic etiology and the need for molecular diagnostic technology development [1, 10]. Four loci located close to genes *ITIH3*, *AS3MT*, *CACNA1C*, and *CACNB2* reached genome-wide significance in meta-analysis of five major psychiatric disorders (SCZ, BD, ASD, ADHD, and depression) in the PGC study with the same direction of effects for these diseases [1]. For three out of the four loci, the five disorders did not show significant difference between each other in a meta-analysis homogeneity test and the model of best fit includes the contribution of all five disorders [1]. An additional seven loci were found approaching genome-wide significance (*P* value < 1 × 10^−6^) and 20 genes in the calcium channel activity pathway were significantly enriched in the dataset of each of the five psychiatric disorders [1]. With the rapid development of high-throughput sequencing technology, a growing catalog of de novo loss-of-function (LoF) mutations have been identified for each of the neuropsychiatric disorders. Statistical analyses have also revealed significant overlap for LoF mutations between neuropsychiatric disorders [11]. For example, LoF mutations in ten genes are shared between ASD and SCZ (*CHD8*, *ZMYND11*, *CRYBG3*, *YTHDC1*, *HIVEP3*, *TNRC18*, *MOV10*, *ST3GAL6*, *PHF7*, *SMARCC2*) [11–14]. Compared to the other neuropsychiatric disorders, depression has been more enigmatic. To date, 23 genome-wide significant loci have been identified from five GWAS on major depressive disorder [15–19]. Among these 23 loci, ten (*PAX5*, *RERE*, *VRK2*, *MEF2C*, *L3MBTL2*, *DCC*, *SORCS3*, *NEGR1*, *VRK2*, *LIN28B*) were shared with other neuropsychiatric disorders (SCZ, BD, ASD, ADHD) reported in the GWAS catalog [20]. Less progress has been made with respect to CNVs. We have observed significant sharing of CNVs across different neurodevelopmental/psychiatric diseases, impacting genes that belong to the metabotropic glutamate receptor gene networks [6, 7, 21]; *CACNA1B* was identified as significant in a schizophrenia case-control CNV study [4], and subsequently a close homolog, *CACNA1C*, was identified as significant by the PGC GWAS [3]. Deletions upstream of *CNTN*4 were identified as significant in an autism CNV study [5] as well as significant in an ADHD study [7]. Others have also found common CNV loci contributing to more than one neuropsychiatric disease. For example, the CNV at the 16p11.2 locus is associated with both SCZ and BD [22], the duplication at 17q12 is shared between ASD, intellectual disability, and SCZ [23], and the CNVs at 15q13.3, 22q11.2, and in the *NRXN1* gene are each associated with a wide spectrum of neurodevelopmental disorders [24–26]. However, no study has systematically examined which rare recurrent shared genetic loci bearing CNVs impact the development of multiple neuropsychiatric disorders.

Here, we analyze five major psychiatric disease cohorts, including 7849 cases and 10,799 controls, in a systematic manner to promote comparability of results, and more importantly to understand the degree to which the shared CNV loci may similarly or differently impact the development of neuropsychiatric disorders.

## Methods

### Study subjects and genotyping

Our study is composed of cases of neurodevelopmental and neuropsychiatric diseases and healthy controls from five independent cohorts (Table 1).

**Table 1:** The neurodevelopmental and neuropsychiatric disease cohorts analyzed after quality control filtering

Disease cohort	Cases	Controls	Array
Janssen SCZ and BD	2917	1113	Illumina 1MDv3
CHOP SCZ	965	1467	Affymetrix 6.0
CHOP ASD	2079	2519	Illumina 550v3
CHOP ADHD	1241	4110	Illumina 550v1
Depression	647	1590	Perlegen 660 k

*SCZ* schizophrenia, *BD* bipolar disorder, *ASD* autism spectrum disorders, *ADHD* attention-deficit hyperactivity disorder

#### Janssen SCZ and BD cohort

The samples, including 3251 schizophrenia, 377 schizoaffective disorder, and 1344 bipolar cases, were collected from 28 clinical trials conducted by Janssen Research & Development, LLC, as described previously [10, 27]. These samples were genotyped on the Illumina 1MDuoV3 array. They were matched to controls, from the biorepository at the Center for Applied Genomics (CAG) of the Children’s Hospital of Philadelphia (CHOP), which were also genotyped on the Illumina 1MDuoV3 arrays to ensure consistency in CNV discovery biases. All controls were recruited at CHOP and had no diagnosis or family history of psychiatric disease based on their medical record. [10]

#### CHOP SCZ cohort

The cases and controls were from CAG at CHOP and the Department of Psychiatry at the University of Pennsylvania, School of Medicine. All cases meet DSM-IV-TR criteria for schizophrenia or schizoaffective disorder [4]. A subject is excluded if he/she is unable to give informed consent to all aspects of the study, or is unable to speak and be interviewed in English, or has severe mental retardation. All samples were genotyped on the Affymetrix 6.0 array at CHOP, as described previously [4].

#### CHOP ASD cohort

The autism cohort included 3360 cases from Autism Genetics Resource exchange (AGRE), Autism Genome Project (AGP), and Autism Cases recruited and genotyped at CAG [5]. The control group included children of self-reported Caucasian ancestry, recruited at CHOP. All controls had no history of ASD, or any other central nervous system disorder, chromosomal disorder, syndrome or genetic disorder. All samples were genotyped on the Illumina HumanHap550 chip [5].

#### CHOP ADHD cohort

The 1013 ADHD cases of European descent were recruited and genotyped at CHOP; additional cases were from NIMH and The University of Utah [7]. The control group included healthy children aged 6–18 years old, with no serious underlying medical disorder, including but not limited to neurodevelopmental disorders, cancer, chromosomal abnormalities, and known metabolic or genetic disorders [7]. Samples were genotyped on the Illumina HumanHap 550 chip [7].

#### Depression cohort

The depression cohort included cases and controls from the Genetic Association Information Network (GAIN) major depressive disorder (MDD)/Netherlands Study of Depression and Anxiety (NESDA) project (phs000020.v2.p1) [28]. The depression cohort cases and controls were genotyped on the Perlegen 660 k array (Perlegen Sciences Mountain View, CA, USA).

### Principal component analysis

PCA was conducted on the SNP genotype using the Eigenstrat [29] package to infer population structure. The first two principal components were plotted to exclude those outliers of non-European ancestry. Only subjects of European ancestry were kept for further association analysis.

### CNV detection

For the Affymetrix 6.0 array, the CEL files were first converted to raw intensity data using our PennCNV [30] Affy workflow (http://penncnv.openbioinformatics.org/en/latest/user-guide/affy/). Then for all array types, CNVs from all samples were generated using PennCNV [30], a hidden Markov model (HMM)-based algorithm which combines multiple sources of information, including log R ratio (LRR), the B allele frequency (BAF) of each SNP, SNP spacing, and population frequency of B allele, to generate CNVs. Only CNVs containing more than three SNPs were generated. As large CNVs tend to be split into small fragments during the CNV calling procedure, adjacent CNV calls were merged via the clean_cnv.pl program implemented in PennCNV with the default parameters.

### Quality control filtering

Sample quality control (QC) steps were performed to remove the related and/or problematic samples. First, gender discrepancies were examined using both the heterozygosity rate of the X-chromosome SNPs and the call rate of the Y-chromosome SNPs. Samples with discrepant and ambiguous gender information were excluded. Second, the relatedness of the genotyped samples was examined using pairwise Identity-by-State analysis via PLINK [31]. Duplicated samples with discrepant phenotype data were excluded from subsequent analyses. For each pair of samples that were duplicates with consistent phenotype data, or samples of related individuals (PI_HAT value exceeding 0.3), the sample with the smaller standard deviation of LRR (LRR SD) was retained.

QC was also conducted on samples based on CNV metrics: genotyping rate, LRR SD indication of intensity noise, |GC base pair wave factor (GCWF)| indication of intensity waviness, and CNV count per sample suggestive of DNA quality.

Because of differences between array types, we reviewed data on each array type separately and plotted the distribution of each CNV metric; these metrics typically show a linear phase (the majority of the samples) but can also show an exponential phase (including in samples with outlier values). We excluded subjects with any CNV metric in the exponential phase. The QC criteria for each cohort based on the distributions of the CNV metrics are shown in (Additional file 1: Table S1).

Next, we performed QC on called CNVs, excluding CNVs < 20 kb in length and those encompassing fewer than ten probes.

All QC steps were performed for cases and controls together in each cohort. About 20% of samples in each cohort were excluded during QC filtering.

### Fisher exact test

Fisher exact test implemented in software ParseCNV [32] was used to conduct the CNV association analysis on each of the individual cohorts CHOP SCZ, CHOP ASD, CHOP ADHD, and depression. It was also used to compare CNV frequency between cases and controls, as described in previous publications [5, 32]. Deletions and duplications were analyzed separately.

### Linear mixed model association analysis

The “--includeped” option in ParseCNV [32] was used to generate the ped files for additional CNV analysis using CNV “genotype” status. CNV was converted to “genotype” status in the following way: 1 1 for CN = 0, 1 2 for CN = 1, and 2 2 for others in the deletion ped file; 1 1 for CN = 4, 1 2 for CN = 3, and 2 2 for others in the duplication ped file. Then the CNV ped files were imported into GEMMA version 0.94 [33], which could correct for residual sample structure and population stratification. The LMM association testing was performed on the cohort of Janssen SCZ and BD, the samples of which came from various clinical trials. The relatedness matrix for genotype was calculated using the -gk 1 option. The matrix file was then imported for univariate linear mixed model (LMM) association, and the -lmm 4 option was used. We calculated Wald test, likelihood ratio test, and score test statistics. Then we used InsertPlinkPvalue program from the ParseCNV [32] package to insert the SNP *P* value generated by GEMMA back into ParseCNV to collapse neighboring SNPs into CNV regions.

### CNV annotation

Because there is no strong consensus in defining a regulatory region and its targeted gene, we focused our analysis on genes with exonic CNVs which are most likely to directly affect the protein product encoded by the genes. The gene(s) that each exonic CNV region resides in was used to annotate each CNV region. The CNV association *P* value from the Fisher exact test or LMM association analysis was assigned to the gene(s). For genes that contain more than one CNV, multiple-testing correction was conducted by taking the lowest *P* value of the CNVs multiplied by the number of CNVs in this gene.

### Meta-analysis

Fixed effect meta-analysis was then carried out on a gene basis using the software METAL [34]. The logarithm of the odds ratio was taken to ensure consistency with Beta for the direction of association considerations.

### Statistical analysis

We tested a total of 5347 genes harboring exonic CNVs in the meta-analysis of deletions and 6684 genes containing exonic CNVs in the meta-analysis of duplications. Therefore, the multiple-testing adjusted significance threshold is 9.4 × 10^−6^ for deletions and 7.5 × 10^−6^ for duplications. The significance of intersection of risk CNV loci between cohorts was computed using the R package SuperExactTest [35].

### CNV quality review

We conducted manual visual review of the BAF and LRR plots of the significant CNV loci. As we are interested in shared risk genetic loci across different neuropsychiatric diseases, we focused on significant genes harboring case-enriched exonic CNVs in at least two cohorts. BAF and LRR plots of each CNV-containing sample for each significant CNV region were generated with the visualize_cnv.pl program implemented in the software Penncnv [30].

### Quantitative PCR assay

Quantitative PCR (qPCR) was performed with the Universal Probe Library (UPL). UPL probes (Roche, Indianapolis, IN, USA) and corresponding primers for five assays across the targeted *DOCK8*/*KANK1* region were selected using the ProbeFinder v2.49 software (Roche, Indianapolis, IN, USA). qPCR was conducted on an ABI Prism™ 7900HT Sequence Detection System (Applied Biosystems, Foster City, CA, USA). For all samples, qPCR reactions were performed in triplicate, each in 10 μl of reaction mixture containing 10 ng genomic DNA, 100 nM of the UPL probe, 400 nM of each PCR primer, and 1× TaqMan Gene Expression Master Mix containing UDG and ROX (Life Technologies, Carlsbad, CA, USA), according to the manufacturer’s protocol. Male and female genomic DNA (Promega, Madison, WI, USA) were included in the analysis as controls with expected normal copy number. Results were evaluated using the Sequence Detection Software v2.4 (Applied Biosystems, Foster City, CA, USA) and further analyzed by the ∆ΔC_T_ method. The GAPDH and SNCA genes were used as internal controls and the geometric mean of their C_T_ values was calculated and used as the reference value for ΔC_T_ calculations. The average of values from Promega male and Promega female genomic DNA (Promega, Madison, WI, USA) was considered the reference 2 N sample for ∆ΔC_T_ calculations. Duplications were determined when the relative copy number value for a specific sample normalized to the reference sample was greater than 1.5.

## Results

To identify shared structural variants underlying the development of neuropsychiatric disorders, we took an unbiased approach based on genome-wide SNP array intensity data from five psychiatric disease cohorts, including SCZ, BD, ASD, ADHD, and depression. After QC filtering based on sample quality and CNV metrics (“Methods”; Additional file 1: Table S1), 7849 cases and 10,799 controls were left for analysis (Table 1). Then we performed case–control association testing on CNVs from each cohort.

Next we performed gene-based meta-analysis. The SNP coverage differs between arrays and one necessary component for CNV calling, the SNP intensity data, cannot be imputed, so there may not be extensive direct overlap between CNVs across different cohorts. We annotated each CNV overlapping a gene exon(s) with the gene(s) in which the CNV region resides. Similar to other types of rare variants, CNVs may reside in different regions of a gene, which is important for brain development. Thus, we took a gene-based approach to allow for more dynamic matching between CNVs. The gene-based association testing method has been frequently used in common-variant and rare-variant analyses [36–42].

In our study, we focused on genes that contain exonic CNVs, which are case-enriched in at least two cohorts. This is because exonic CNVs are most likely to directly affect the protein product encoded by the genes and there is no strong consensus in defining a regulatory region and its targeted gene. Another consideration is the direction of effects that each CNV gives rise to. Though control-enriched CNVs may have potential protective effects, e.g. those of the 22q11.2 CNV region against schizophrenia as reported by Rees et al. [43], this is still actively debated in the CNV study field, and the mechanism by which 22q11.2 CNVs affect the risk of disease development is under investigation. We favor the deleterious model of CNVs, consistent with the field’s standard and the focus of our study is risk CNVs shared by neuropsychiatric disorders. Therefore, we kept only significant exonic CNVs that are case-enriched in at least two cohorts. We observed significant overlap of such CNV-containing genes between cohorts (Additional file 2: Figure S1). Two genes (*DOCK8* and *LOC100131257*) were found in all five cohorts (*P* value = 8.29 × 10^−6^).

**Fig. 1:**
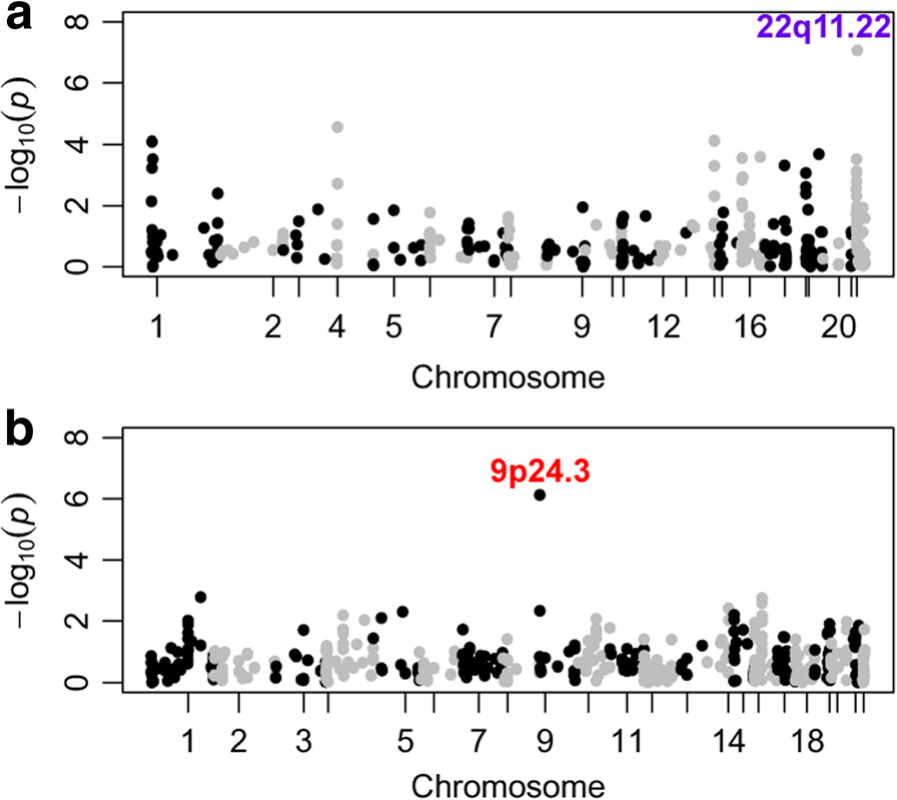
Manhattan plots for gene-based CNV meta-analysis. The results for deletion CNVs are shown in **a** and those for duplication CNVs are shown in **b**. The − log10(*P* value) of each gene (y-axis) in the meta-analysis is plotted against the genomic position (x-axis). Significant loci are indicated on the plot. The locus of 22q11.22 is a known locus for neuropsychiatric disorders and 9p24.3 is a novel locus at which each CNV carrier has been validated by manual visual review of BAF and LRR plots and qPCR experiments

In the meta-analysis, the *ZNF280A* and *DOCK8* genes reached a genome-wide significance threshold of 9.4 × 10^−6^ for deletions and 7.5 × 10^−6^ for duplications (Table 2, Fig. 1) and passed the manual review of their BAF and LRR plots for each CNV carrier sample (Additional file 2: Figure S2 and S3). *KANK1*, which is next to *DOCK8* and contains exonic duplications, is of suggestive significance (*P* = 3.45 × 10^−5^). The locus of 22q11.22, encompassing the *ZNF280A* gene, is a known risk CNV locus for SCZ and BD [44]. Deletions overlapping the *ZNF280A* gene locus were observed in four cohorts. It is significantly case-enriched for the CHOP ADHD and ASD cohorts, in which 22q11.22 deletion was only observed among cases and not among controls, but this locus is control-enriched in the depression and Janssen SCZ and BD cohorts (Table 2, Fig. 1)

**Table 2:** Significant loci in gene-based meta-analysis of the five neurodevelopmental/neuropsychiatric cohorts that contain case-enriched exonic CNVs in two or more cohorts

Marker name	Cytoband	CNV type	Number of cohorts observed	Meta *P* value	Direction of effect
*ZNF280A*	22q11.22*	Del	4	8.63E-08	-?++-
*DOCK8*	9p24.3	Dup	5	7.50E-07	+++++

The *Number of cohorts* is the number of cohorts in which CNVs were observed overlapping exons of the gene. The *Direction of effect* is reported in the order of Janssen SCZ and BD, CHOP SCZ, CHOP ASD, CHOP ADHD, and depression cohorts: a plus sign means CNVs are enriched in cases, a dash means CNVs are enriched in controls, a question mark means no CNV is observed overlapping the exons of this gene. *Del* deletion, *Dup* duplication.

* Known neuropsychiatric-disorder-associated regions

**Fig. 2:**
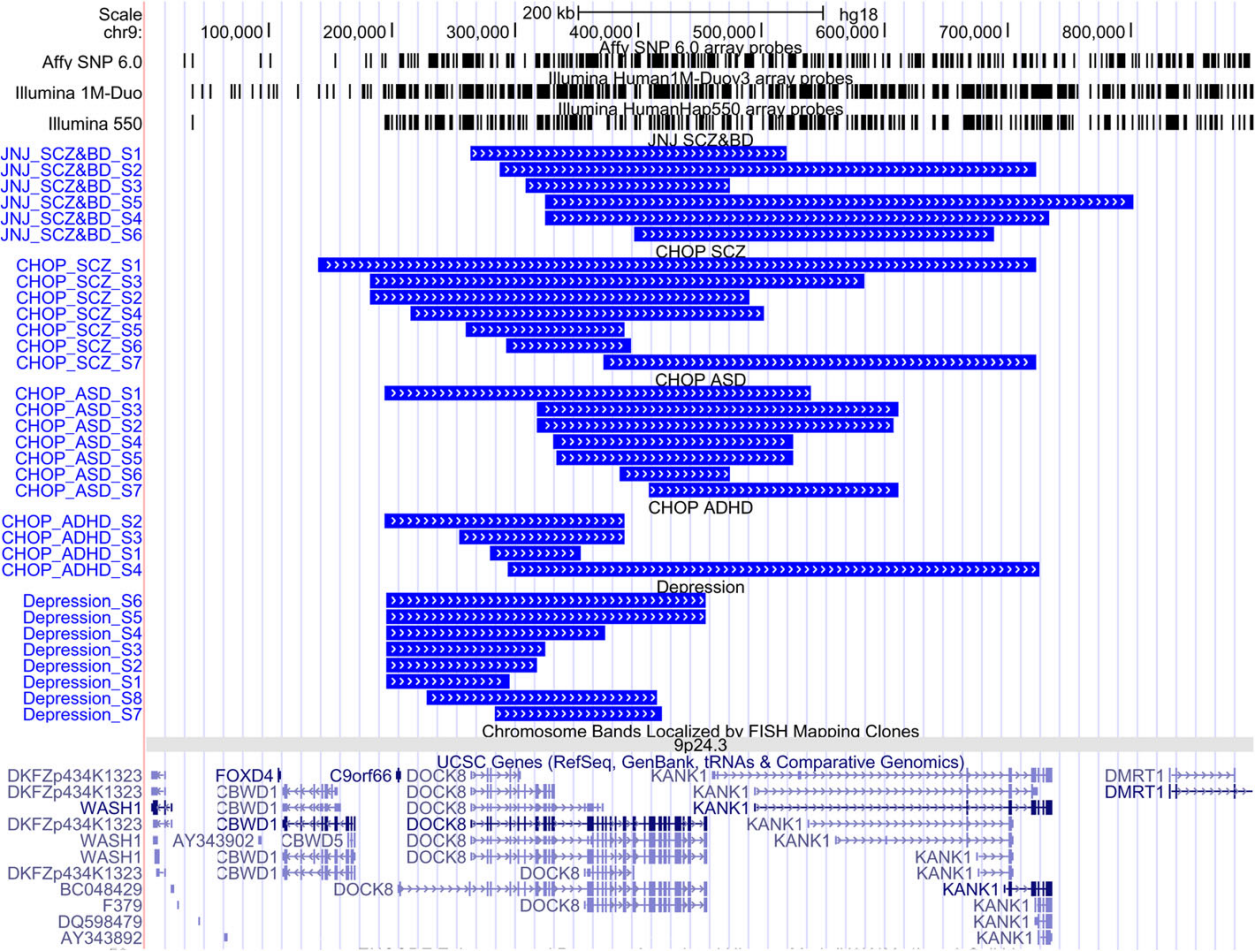
Contributing calls in the *DOCK8*/*KANK1* region from each cohort. *Black bars* indicate the SNP coverage of each genotyping array and *blue rectangles* represent each individual duplication call observed among neuropsychiatric cases in each cohort

We identified a novel CNV locus demonstrating significant association across the neuropsychiatric disorders under study. The *DOCK8* gene at cytoband 9p24.3 showed significant association with neuropsychiatric disorders in meta-analysis. *DOCK8* and the neighboring gene *KANK1* exhibited significant or marginally significant case enrichment in all five cohorts (Tables 2 and 3, Fig. 2), especially in the Janssen SCZ and BD cohort as well as the CHOP ASD cohort, in which more than 5 cases but no controls carry CNVs in this region (Table 3). As the samples in the Janssen SCZ and BD cohort were from diverse resources and were not genotyped at CHOP, to ensure CNV quality at this region, we specifically conducted validation for CNV carriers in this cohort by two approaches. First, we manually examined the CNV calls by visually reviewing the raw intensity and genotype values for probes in this region and flanking regions (Additional file 2: Figure S3). We have previously reported that visually validated CNVs yield experimental validation success rates above 95% [32]. For the CNVs contributing calls in this region, we observed a moderate gain in LRR intensity and alternative banding of BAF at 0.33 and 0.66 instead of 0.5, which are indicative of clear duplication CNV signals. Second, we also tested the CNV status of this region using an independent experimental approach, the qPCR assay, on the above samples (Table 4). We included one sample from our cohort without a CNV in this region as a negative control. Each sample containing a duplication in the *DOCK8–KANK1* region was tested by five qPCR assays. The results showed that the CNV in each of the ten samples was also detected by the corresponding qPCR assays, and the CN = 2 control sample did not show any CNV in all qPCR assays across this region. There were a few regions flanking the CNVs detected by arrays where duplications were observed with qPCR, refining the CNV boundaries. Thus, the CNV data from the arrays were validated by both an in silico approach and an independent experimental assay. An inconsistency between the array and qPCR results was seen for sample S9 with probe 141, and the array result for this sample is likely due to ambiguity in CNV boundary determination rather than presence vs. absence based on CNV calling from SNP arrays.

**Table 3:** Contributing signals for the *DOCK8* gene from each psychiatric disease cohort

Disease cohort	Overlapping CNV region (hg18)	CNV type	Odds ratio	*P* value	Number of case CNVs (%)	Number of control CNVs (%)
Janssen SCZ and BD	Chr9:396118-474850	Dup	Infinity	0.00693	6 (0.21)	0 (0)
CHOP SCZ	Chr9:372245-389052	Dup	10.71	0.008	7 (0.73)	1 (0.07)
CHOP ASD	Chr9:407918-474786	Dup	Infinity	0.00384	7 (0.34)	0 (0)
CHOP ADHD	Chr9: 293639-352917	Dup	3.32	0.0899	4 (0.32)	4 (0.10)
Depression	Chr9: 283360-294957	Dup	4.96	0.00731	8 (1.24)	4 (0.25)

*Number of case CNVs* is the number of cases having a *DOCK8* duplication and the frequency among all cases. *Number of control CNVs* is the number of controls having a *DOCK8* duplication CNV and the frequency among all controls

**Table 4:**
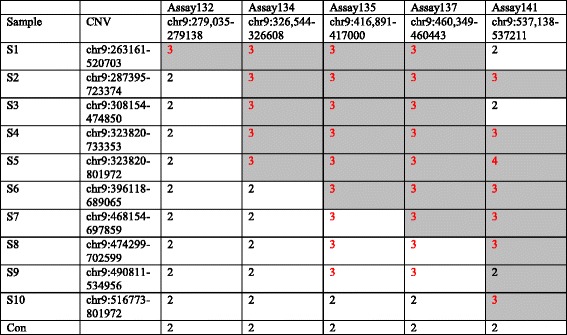
qPCR validation of duplications in the *DOCK8*–*KANK1* region

Each sample was tested by five qPCR assays which cover much of the duplication regions. Each result represents copy number calculated from triplicate runs. The table has been shaded *gray* for assays that were within the duplication call for that subject detected by arrays, and the CNVs validated by qPCR are highlighted in *red*

Each of the contributing CNVs overlapping regions from the five cohorts overlaps with *DOCK8* exons, which is likely to have an impact on *DOCK8* expression. In addition, assessing the annotations in ENCODE [45] and ROADMAP [46] databases, we found that the *DOCK8* overlapping CNV region from each cohort contains many histone marks and DNase sites (Additional file 1: Table S2). Further interrogating these regions in two eQTL databases—GTEx [47] and GRASP [48]—we observed significant brain tissue eQTL SNPs in *DOCK8* overlapping the CNV region in four out of the five cohorts (Additional file 1: Table S2). For the significant eQTLs, the genes regulated include *CBWD1*, *DMRT3*, *DOCK8*, *FOXD4*, and *KANK1*, all of which are located in the same topologically associating domains (TADs; Additional file 2: Figure S4), suggesting that the expression of these genes may also be affected.

## Discussion

Cumulative evidence indicates a shared genetic etiology of neurodevelopmental and neuropsychiatric diseases. We performed CNV meta-analysis in five major neurodevelopmental/psychiatric diseases. Using gene-based association statistics, we robustly meta-analyzed different psychiatric conditions across distinct microarrays. This is the first large-scale CNV meta-analysis across a spectrum of neuropsychiatric disorders. We identified the *DOCK8*/*KANK1* locus as containing exonic CNVs with genome-wide significant meta *P* values and consistent direction of effects across all five cohorts. The highly significant associations of *DOCK8*/*KANK1* duplications were further validated by an independent experimental approach. The identification of shared structural variants underlying the five neuropsychiatric disorders helps to refine the genetic basis for co-morbidity and co-occurrence of neuropsychiatric diseases among individuals or families has the potential to help in the development of common therapeutics of shared genetic targets across different diseases.

The duplications of *DOCK8* and *KANK1* at 9p24.3 are intriguing given that these genes have been shown to be involved in neurodevelopment and neurological functions. *DOCK8* is the dedicator of cytokinesis 8, a member of the DOCK180 family of guanine nucleotide exchange factors (GEF), which includes 11 DOCK genes [49, 50]. GEF proteins are important components of intracellular signaling networks, activating small GTPases by exchanging bound GDP for free GTP [51]. *DOCK8* is expressed in adult and fetal brain tissues and deletion or translocation breakpoints that disrupt its function have been found in individual patients with intellectual disability [52]. This is the first time that *DOCK8* duplications were found to be significantly associated with a spectrum of neurospsychiatric disorders, suggesting that a tightly regulated *DOCK8* expression level may be required for normal cellular function. The neighboring gene of *DOCK8* is *KANK1* (KN motif and ankyrin repeat domains 1), which has also been demonstrated to play a role in neuronal functions. KANK1 functions in actin cytoskeleton formation by competing for 14-3-3 binding upon phosphorylation by Akt and inhibiting RhoA activity [53, 54]. It inhibits neurite outgrowth, actin fiber formation, and cell migration, depending on the competitive interaction with BAIAP2 to block its association with activated RAC1 [55]. KANK1 functions in regulating microtubule dynamics at the cell cortex by recruiting KIF21A, which is important in neuronal development [56, 57]. Mutations in this gene cause cerebral palsy spastic quadriplegic type 2, a central nervous system development disorder [58]. In the DECIPHER dataset, 16 subjects with intellectual disability carry *DOCK8* duplications/gains and ten carry *KANK1* duplications/gains [59].

The novelty of our finding lies in the illustration of the significant association of *DOCK8*/*KANK1* with multiple neuropsychiatric diseases. In the CNV study of Coe et al. [60] for intellectual disability, developmental delay, and/or ASD, *DOCK8* is nominally significant for deletions (*P* = 0.000281) but not duplications. In the recent CNV study of schizophrenia by Marshall et al. [42], *DMRT1*, which is at the same cytoband of 9p24.3, was reported to be a novel CNV-containing gene significantly associated with schizophrenia in the test for a combined (deletion + duplication) CNV analysis. The reported *DMRT1* CNV locus (chr9:831690–959090, hg18) is more than 400 kb downstream of the *DOCK8*/*KANK1* region found in our study. In addition, the *DOCK8*/*KANK1* region is not highly prone to CNV in the general population. In our study, 0.25% or less of the control subjects carry *DOCK8*/*KANK1* duplications in each cohort. The Database of Genomic Variants (DGV) [61] shows 65 duplications in the *DOCK8* gene. Among them, 33 were reported in CNV studies of developmental delay [60, 62], and 32 duplications in *DOCK8* were reported from other CNV population studies. This is also similar to another gene, *NRXN1*, the deletion of which is significantly associated with schizophrenia [63]. A total of 144 deletions/loss were reported in DGV for the *NRXN1* gene, among which 45 were reported in the CNV studies of developmental delay and the remaining 99 were from other CNV population studies. Therefore, the significant association in the meta-analysis reflects its potential contribution to the pathology of neurodevelopmental/psychiatric disorders. In addition, the novelty of our finding lies in the identification of the significant association of *DOCK8* duplication with multiple neurodevelopmental/psychiatric disorders across cohorts (association *P* < 0.01 for four out of five cohorts), implicating its common role as a risk locus for these diseases.

To compensate for the incomplete overlapping in SNP coverage between study cohorts, we conducted gene-based meta-analysis, which has been widely adopted in both common and rare variant analyses. One caveat is that CNV overlaps with different gene regions might not have the same effect on the expression of the gene. Some may result in frame shifts and the complete loss of function of the gene and some may have minor effects on the gene. Nevertheless, the biological function of each gene is maintained in a fine balanced state for cellular activities. Even minor perturbation of its expression could lead to pathological consequences. The discovery of CNVs in *DOCK8* and *KANK1* across all five diseases not only adds to the growing catalog of neurodevelopmental variants but also paves the way for new diagnostics opportunities and interventions which could be applied across multiple clinical indications. However, functional studies are needed to better understand the biological effect of these variations.

## Conclusions

With the growing awareness of the high impact of childhood psychiatric conditions comes the important need for large-scale genetic studies and a unified picture of the catalog of rare variants underlying these conditions. We have undertaken the unprecedented step to meta-analyze CNVs across five neurodevelopmental/psychiatric diseases and have uncovered significant structural variation at the *DOCK8*/*KANK1* locus shared by these diseases, emphasizing the common genetic component involved in the pathogenesis of neuropsychiatric disorders.

## Additional files

Additional file 1: Table S1. The quality control criteria based on CNV metrics applied to each cohort. **Table S2.** The number of histone marks, DNase, and eQTLs in each *DOCK8* CNV. (PDF 127 kb)

Additional file 2: **Figure S1.** Circular plot showing the intersections of genes harboring case-enriched CNVs between the five neuropsychiatric cohorts. **Figure S2.** The BAF and LRR plots of *ZNF280A* deletions. **Figure S3.** The BAF and LRR plots of *DOCK8*/*KANK1* duplications. **Figure S4.** The topologically associating domains (TAD) at the chromosome 9 *DOCK8*/*KANK1* region. (PDF 2076 kb)

## Abbreviations

ADHD: Attention deficit hyperactivity disorder
AGP: Autism Genome Project
AGRE: Autism Genetics Resource Exchange
ASD: Autism spectrum disorders
BAF: B allele frequency
BD: Bipolar disease
CAG: Center for Applied Genomics
CHOP: the Children’s Hospital of Philadelphia
CNV: Copy number variation
GAIN: Genetic Association Information Network
GCWF: GC base pair wave factor
GEF: Guanine nucleotide exchange factor
GWAS: Genome-wide association studies
KANK1: KN motif and ankyrin repeat domains 1
LMM: Linear mixed model
LRR: Log R ratio
MDD: Major depressive disorder
NESDA: Netherlands Study of Depression and Anxiety
PCA: Principal component analysis
QC: Quality control
qPCR: Quantitative polymerase chain reaction
SCZ: Schizophrenia
SNP: Single nucleotide polymorphism
TAD: Topologically associating domains
UPL: Universal Probe Library
