# Supplementary material for: Copy number variation meta-analysis reveals a novel duplication at 9p24 associated with multiple neurodevelopmental disorders
Source: Genome Med. 2017 Nov 30;9:106. doi: 10.1186/s13073-017-0494-1 (PMC5709845; doi:10.1186/s13073-017-0494-1)
Supplement: Supplementary file 1 — The quality control criteria based on CNV metrics applied to each cohort. Table S2. The number of histone marks, DNase, and eQTLs in each DOCK8 CNV. (PDF 127 kb) [file 13073_2017_494_MOESM1_ESM.pdf]

Supplemental Tables:

Table S1. The quality control criteria based on CNV metrics applied to each cohort.

Disease Cohort	genotyping rate	LRR SD	|GCWF|	CNV count
**Janssen SCZ & BD**	>98%	<0.3	<0.05	<100
**CHOP SCZ**	>96%	<0.35	<0.02	<80
**CHOP ASD**	>98%	<0.3	<0.05	<100
**CHOP ADHD**	>98%	<0.3	<0.05	<100
**Depression**	>98%	<0.25	<0.02	<100

LRR SD= the SD of log R ratio; |GCWF|= the absolute value of GC base pair wave factor; CNV count= CNV count per sample.

Table S2. The number of histone marks, DNase and eQTLs in each *DOCK8* CNVs.

Disease Cohort	CNVR (hg18)	H3K4me1 Enh	H3K4me3 Pro	H3K27ac Enh	H3K9ac Pro	Dnase	GTEx	GRASP
**JANSSEN SCZ & BD**	chr9:396118-474850	269	102	204	110	57	1	1
**CHOP SCZ**	chr9:372245-389052	303	120	204	110	59	1	1
**CHOP ASD**	chr9:407918-474786	151	32	158	161	17	0	0
**CHOP ADHD**	chr9: 293639-352917	276	71	167	221	45	2	1
**Depression**	chr9: 283360-294957	92	39	63	0	12	1	0
