# Supplementary material for: Copy number variation meta-analysis reveals a novel duplication at 9p24 associated with multiple neurodevelopmental disorders
Source: Genome Med. 2017 Nov 30;9:106. doi: 10.1186/s13073-017-0494-1 (PMC5709845; doi:10.1186/s13073-017-0494-1)

Supplemental Figures:

Figure S1. Circular plot showing the intersections of genes harboring case-enriched CNVs between the five neuropsychiatric cohorts.

The five tracks in the middle of the circular plot indicates the five neuropsychiatric cohorts, and the color of each individual block represents the inclusion (green) or exclusion (grey) of the cohorts in each combination. The height of the bars in the outmost layer represents the intersection sizes with the actual number shown next to the bars; and the color of the bars suggests the statistical significance of each intersection.

Set 1: JANSSEN SCZ & BD, Set 2: CHOP SCZ, Set 3: CHOP ASD, Set 4: CHOP ADHD, Set 5: Depression

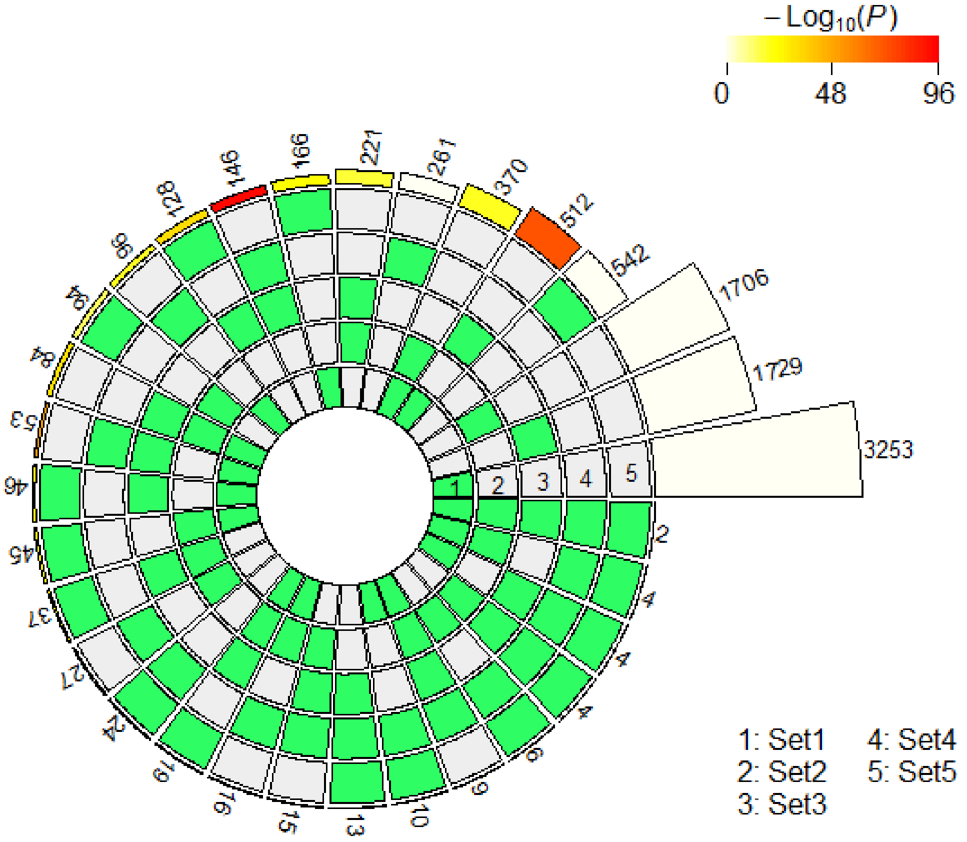

Figure S2. The BAF LRR Plots of *ZNF280A* deletions.

The Log R ratio (LRR) plot and the B allele frequency (BAF) plot of *ZNF280A* deletions in 9 representative samples are shown. The LRR plot is shown on the top and the BAF plot is shown on the bottom for each sample.

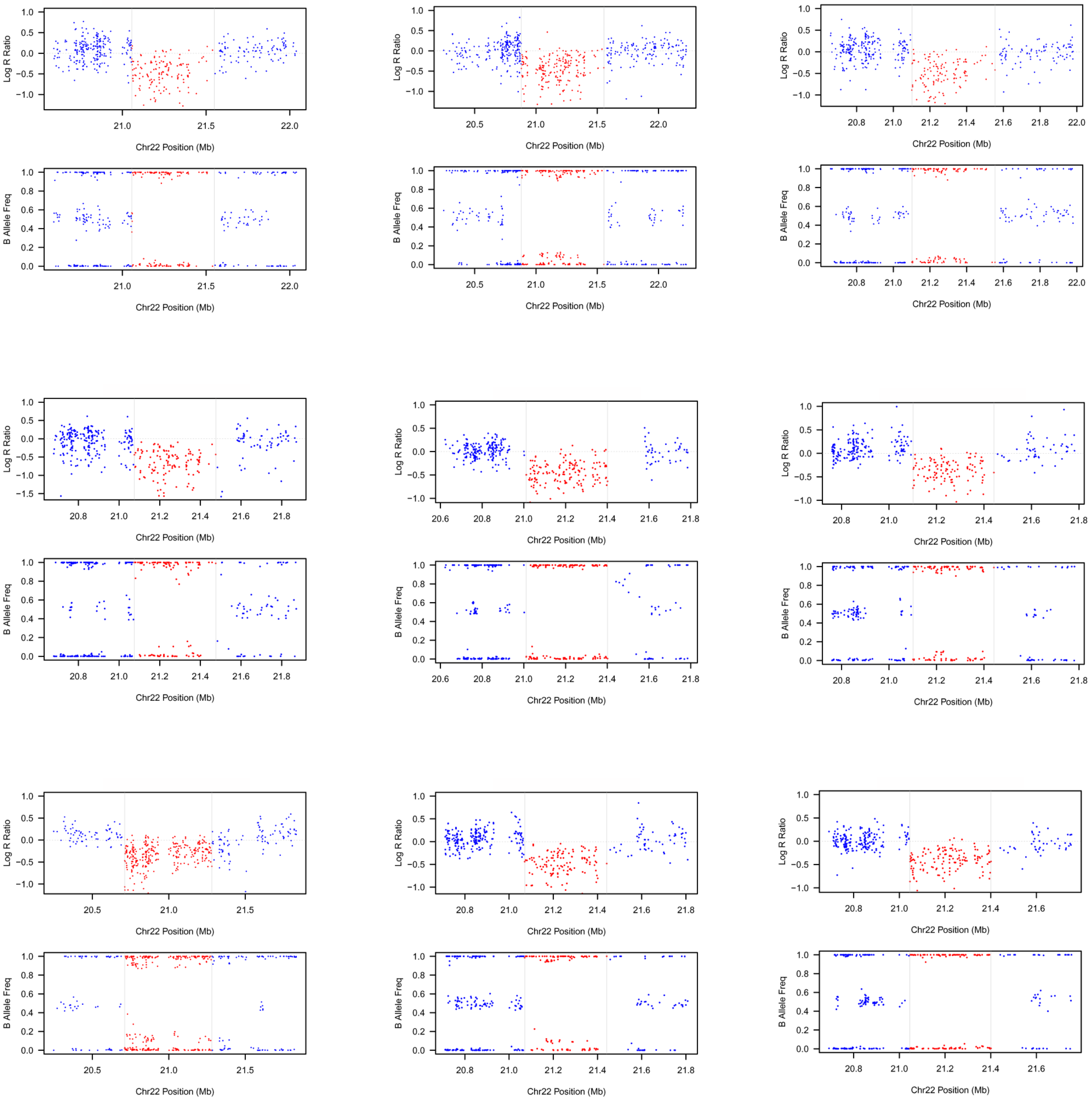

Figure S3. The BAF LRR Plots of *DOCK8/KANK1* duplications.

Samples are indicated as S1-S9. The Log R ratio (LRR) plot is shown on the top and the B allele frequency (BAF) plot is shown on the bottom for each sample.

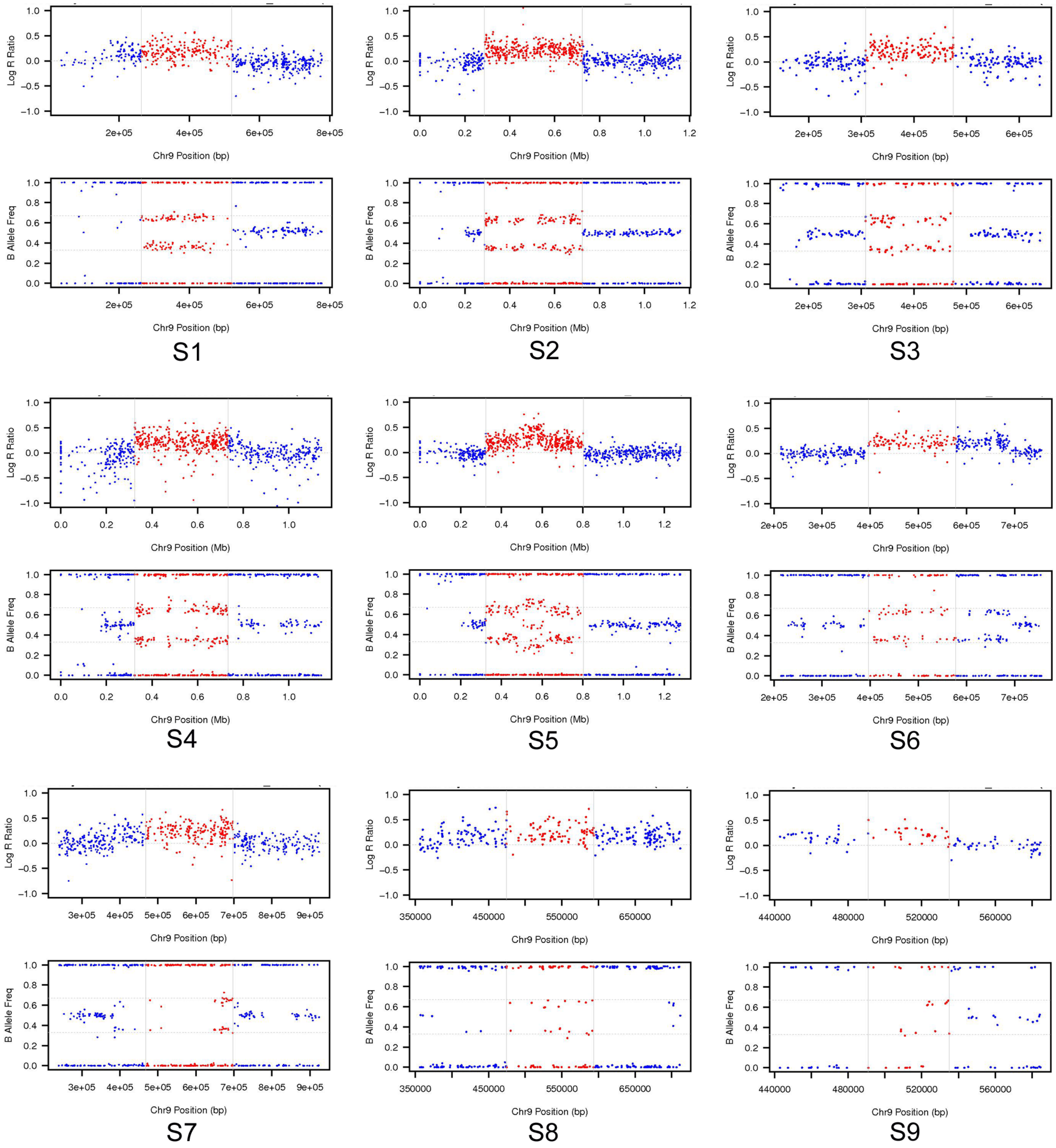

Figure S4. The topologically associating domains (TAD) at on chromosome 9 *DOCK8/KANK1* region. Heatmap shows pairwise interaction frequencies.

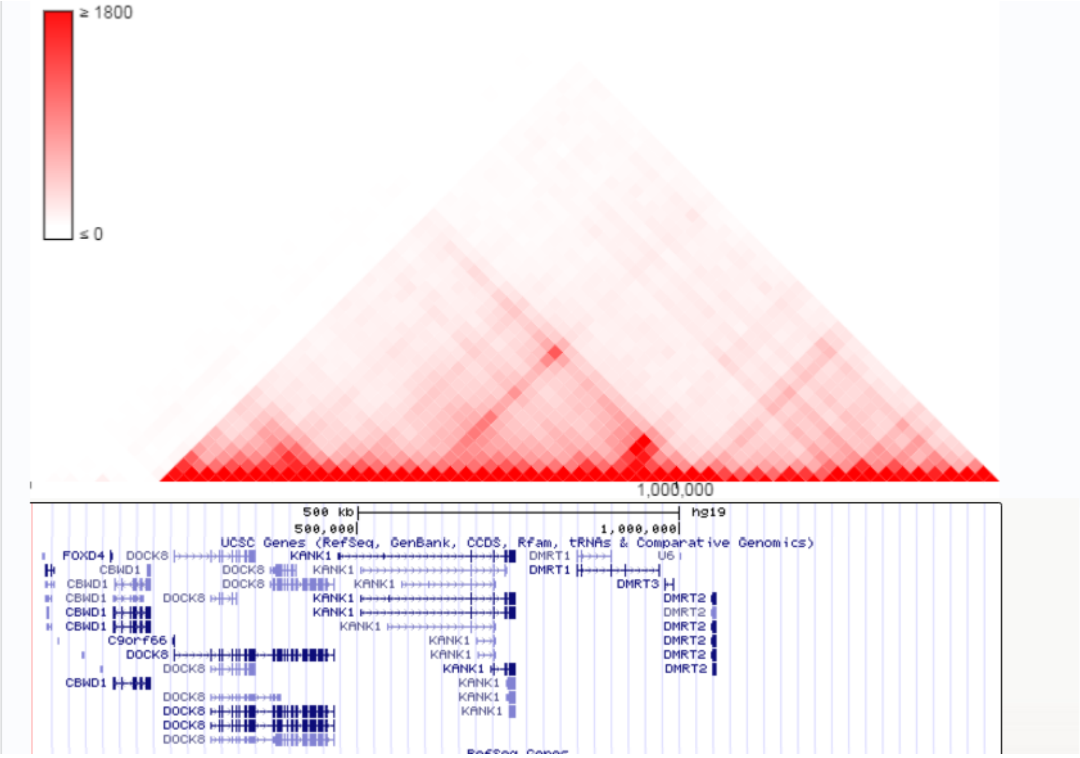

Supplement: Supplementary file 2 — Figure S1. Circular plot showing the intersections of genes harboring case-enriched CNVs between the five neuropsychiatric cohorts. Figure S2. The BAF and LRR plots of ZNF280A deletions. Figure S3. The BAF and LRR plots of DOCK8/KANK1 duplications. Figure S4. The topologically associating domains (TAD) at the chromosome 9 DOCK8/KANK1 region. (PDF 2076 kb) [file 13073_2017_494_MOESM2_ESM.pdf]
